# Wedge-shaped vertebrae is a risk factor for symptomatic upper lumbar disc herniation

**DOI:** 10.1186/s13018-019-1314-7

**Published:** 2019-08-22

**Authors:** Feng Wang, Zhen Dong, Yi-peng Li, De-chao Miao, Lin-feng Wang, Yong Shen

**Affiliations:** 1 Department of Spine Surgery, The Key Laboratory of Orthopedic Biomechanics of Hebei Province, The Third Hospital of Hebei Medical University, 139 Ziqiang Road, Shijiazhuang, 050000 China; 2 Department of Orthopedics, Shijiazhuang No.1 Hospital, 36 Fanxi Road, Shijiazhuang, 050000 China

**Keywords:** Upper lumbar disc herniation, Lower lumbar disc herniation, Wedge-shaped vertebrae, Lumbar lordosis

## Abstract

**Background:**

At present, much is unknown about the etiology and pathogenesis of ULDH. However, it is interesting to note that many ULDH patients have a radiographic feature of adjacent vertebral wedge deformation. The purpose of this study is to investigate the relationship between symptomatic upper lumbar disc herniation (ULDH) and wedge-shaped vertebrae (WSV).

**Methods:**

This was a retrospective study of 65 patients with single-level ULDH, who had undergone surgery at our medical center between January 2012 and December 2016. Clinical data including clinical and radiological evaluation results were performed.

**Results:**

The incidence of WSV in the ULDH group (44.6%, 29/65) was more than in the lower lumbar disc herniation group (21.5%, 14/65). And there were statistically significant differences in WSV (*χ*^2^ = 7.819, *P* = 0.005), wedging angle of the vertebrae (WAV) (*t* = 9.013, *P* < 0.001), and thoracolumbar kyphotic angle (TL) (*t* = 8.618, *P* < 0.001) between two groups. Based on multivariate logistic regression analysis, WAV (OR = 0.783, 95% CI = 0.687–0.893, *P* < 0.001) and TL (OR = 0.831, 95% CI = 0.746–0.925, *P* = 0.001) were independently associated with ULDH. The cutoff values of WAV and TL were 5.35° and 8.35°, which were significantly associated with ULDH (OR = 3.667, 95% CI = 1.588–8.466, *P* = 0.002).

**Conclusion:**

The WSV is an independent risk factor for ULDH. WAV > 5.35° and TL > 8.35° were the predictors for ULDH. It should be noted that the patients with vertebral wedge deformation combined with thoracolumbar kyphosis have a higher risk of ULDH.

## Background

Lumbar disc herniation (LDH) is defined as a prolapse of the nucleus pulposus from a defect in the annulus fibrosus forming the circumferential rim of the disc. Most LDH occurs at the levels of L4/5 and L5/S1 (90–97%). L1/2 and L2/3 disc herniation, which defined as upper lumbar disc herniation (ULDH), are very rare (< 5%) [1, 2]. ULDH may have different clinical signs than ordinary lower lumbar disc herniation (LLDH) at the levels from L3/4 to L5/S1 in clinical practice. And high rate of neurological disability has been noted in patients with ULDH, and its surgical results differ significantly from those of LLDH [3–5]. To the best of our knowledge, at present, much is unknown about the etiology and pathogenesis of symptomatic ULDH.

It is generally known that the vertebral shape is a major factor in determining the general configuration of the spinal column. We noted that numerous symptomatic ULDH patients visiting our institution had adjacent vertebral wedge-shaped deformities. Although symptomatic ULDH in the context of wedge-shaped vertebrae (WSV) has been recognized to occur, it is still controversial and limited number of cases reported made it difficult to judge the relationship between the ULDH and WSV [6–8].

In this study, a retrospective radiographic review was conducted on 65 symptomatic ULDH patients to investigate the relationship between ULDH and WSV by examining the incidences of associated WSV and its radiologic signs in the ULDH patients from January 2012 to December 2016. And another group of 65 LLDH patients served as controls. We designed the present study to examine the relationship between predictors and ULDH, particularly the WSV. This exploration of the causes of ULDH provided insight for the diagnosis by spine surgeons.

## Materials and methods

### Study population selection

This was a retrospective clinical study. A total of 79 patients underwent single-level posterior lumbar interbody fusion (PLIF) surgery after a diagnosis of symptomatic ULDH (L1/2 or L2/3) at our department between January 2012 and December 2016. Among them, 14 patients who had previous spinal surgery or incomplete radiographic materials were excluded. Finally, 65 patients were enrolled as the ULDH group. There were 33 males and 32 females with a mean age of 42.2 (23–61) years. All patients had neurologic symptoms that warranted surgery. Furthermore, these patients who developed gradual neurological changes followed 6 months of unsuccessful conservative treatment. However, the patients with spine trauma, tumor spinal pathologies, neoplasm, spinal infections, congenital deformations, and chronic systemic illnesses such as rheumatoid arthritis and neurodegenerative diseases were excluded from this study. Data from these ULDH patients were compared with a group of controls that presented with LLDH. They were randomly sampled patients surgically treated (percutaneous endoscopic lumbar discectomy, PELD) in the same period for single-level symptomatic LLDH (L4/5 or L5/S1). The sample size was set at 65 cases in the LLDH group because there were 65 patients in the ULDH group. This study had been approved by Ethics Committee of The Third Hospital of Hebei Medical University. There is no need to obtain informed consent from patients because this is a retrospective study and all data were collected and analyzed anonymously.

### Evaluation criteria

**Fig. 1:**
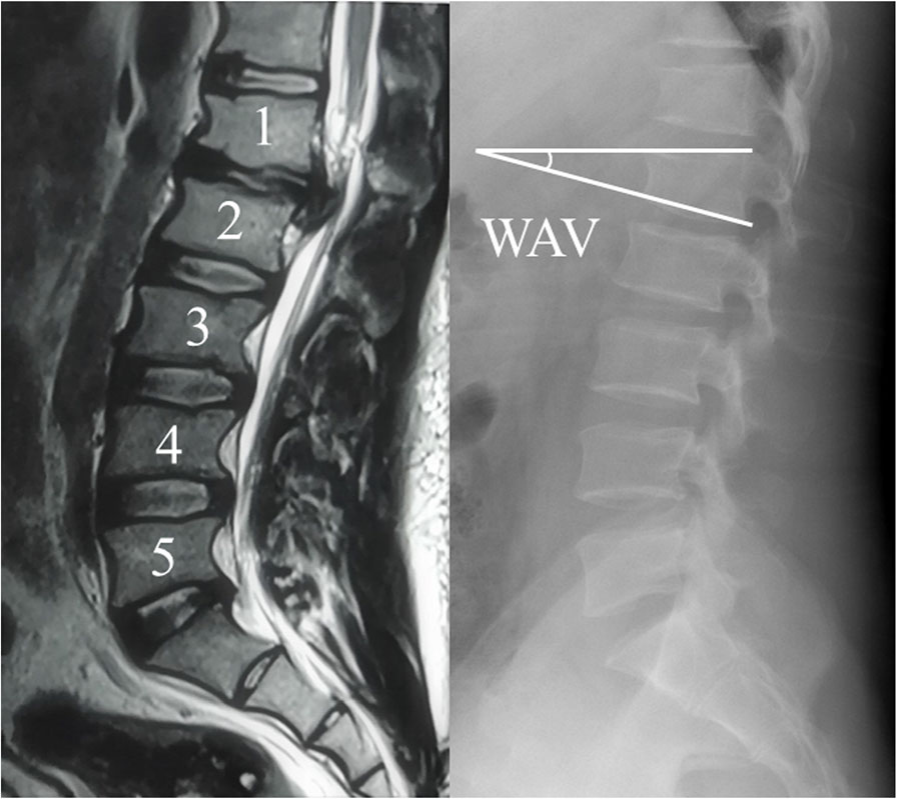
Wedging angle of the vertebrae (WAV) was defined as the angle from the superior endplate to the inferior endplate of the wedge-shaped vertebrae (WSV)

Clinical data including clinical and radiological evaluation results were collected by two independent authors pre- and postoperatively. The thoracolumbar kyphotic angle (TL) was measured from the T10 superior endplate to the L2 inferior endplate by the Cobb method, and lumbar lordosis (LL) was measured from the L1 superior endplate to the S1 superior endplate. In this study, the wedge-shaped vertebrae (WSV) show at least 5° of anterior wedging on the lateral X-ray. And wedging angle of the vertebrae (WAV) was defined as the larger angle adjacent to the herniated disc formed between a line drawn parallel to the superior endplate and a line drawn parallel to the inferior endplate (Fig. 1). In the LLDH group, WAV was measured at each vertebral body from L1 to L3 of every subject and the biggest angle was chosen for study. Two independent radiologists assessed the radiographs. In the event of disagreement about fusion healing, a third independent reading was obtained.

### Statistical analysis

All data were collected, and the software SPSS version 17.0 (SPSS Inc., Chicago, IL) was used for the statistical evaluation. Results were presented as mean ± SD. The independent two-sample *t* test was used to identify a significant difference between two groups. Categorical data were compared via the chi-square test. Multivariate logistic regression analysis was used to predict the risk factors, and *P* value < 0.05 was set for univariate analyses. *P* values of respective predictors were given on the basis of adjusted odds ratio (OR) with 95% confidence interval (CI). The analysis of receiver operating characteristic (ROC) curves was protracted to evaluate the cutoff values for the continuous variables. The relationship between ULDH and the number of risk factors was examined by logistic regression analysis. In all analyses, *P* value < 0.05 was considered statistically significant.

## Results

**Fig. 2:**
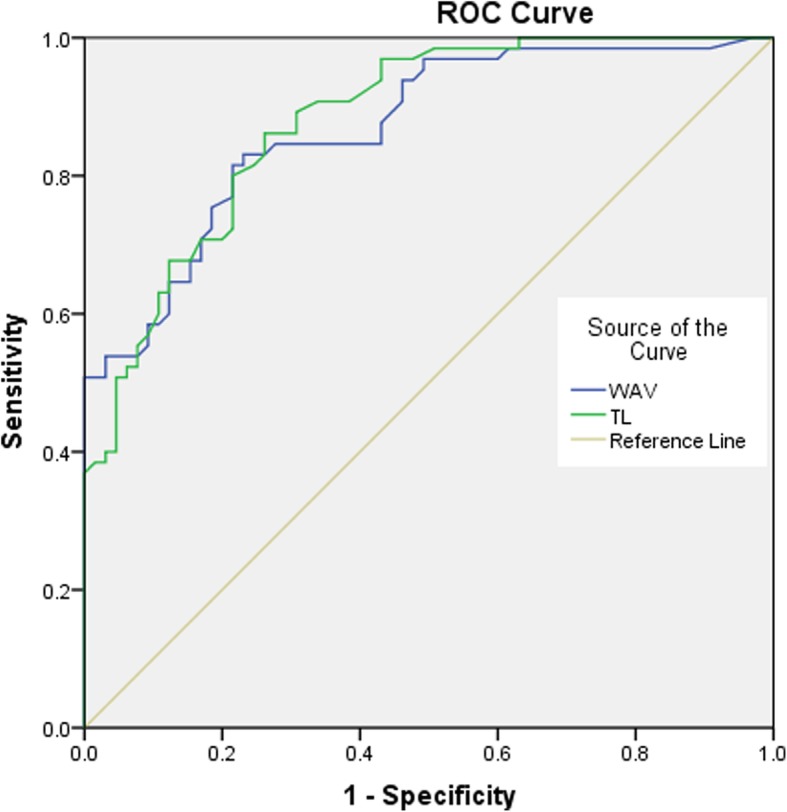
The receiver operating characteristic (ROC) curve of wedging angle of the vertebrae (WAV) and thoracolumbar kyphotic angle (TL)

The univariate analysis showed that there were no significant differences in age at operation, sex, duration of disease, BMI, history of trauma, and LL between the ULDH and LLDH groups (*P* > 0.05) (Table 1). The incidence of WSV in the ULDH group (44.6%, 29/65) was more than in the LLDH group (21.5%, 14/65). And there were statistically significant differences in WSV (*χ*^2^ = 7.819, *P* = 0.005), WAV (*t* = 9.013, *P* < 0.001), and TL (*t* = 8.618, *P* < 0.001) between the ULDH and LLDH groups (Table 1). The variables of WAV and TL were included in a logistic regression model. Based on multivariate logistic regression analysis, WAV (OR = 0.783, 95% CI = 0.687–0.893, *P* < 0.001) and TL (OR = 0.831, 95% CI = 0.746–0.925, *P* = 0.001) were independently associated with ULDH (Table 2). Table 3 and Fig. 2 summarize the relationship for predicting ULDH by the specificity, sensitivity, area under the curve (AUC), and cutoff of risk factors and the receiver operating characteristic (ROC) curve. Furthermore, AUC analysis showed that WAV (AUC = 0.868, *P* < 0.001) and TL (AUC = 0.880, *P* < 0.001) showed good predictive accuracy for ULDH in the ROC curve analysis (Table 3, Fig. 2). The cutoff values of WAV and TL were 5.35° and 8.35°, respectively (Table 3). The presence of two factors (WAV > 5.35° and TL > 8.35°) was significantly associated with ULDH (OR = 3.667, 95% CI = 1.588–8.466, *P* = 0.002) (Table 4).

**Table 1:** Comparison of patient characteristics between ULDH and LLDH groups

Variable	ULDH (65 cases)	LLDH (65 cases)	*t*/*χ*^2^ value	*P* value
Age at operation (years)^a^	42.2 ± 8.0	43.8 ± 7.5	1.176	0.242
Sex^b^				
Male	33	39	1.121	0.290
Female	32	26		
Duration of disease (months)^a^	26.7 ± 15.7	28.6 ± 16.5	0.673	0.502
BMI^a^	25.0 ± 7.4	25.7 ± 6.2	0.585	0.560
History of trauma^b^				
Yes	17	19	0.154	0.695
No	48	46		
WSV^b^				
Yes	29	14	7.819	0.005^*^
No	36	51		
WAV^a^	11.2 ± 6.2	3.4 ± 3.2	9.013	< 0.001^*^
TL^a^	16.3 ± 8.2	6.5 ± 4.1	8.618	< 0.001^*^
LL^a^	36.6 ± 8.2	39.1 ± 7.6	1.802	0.074

*ULDH* upper lumbar disc herniation, *LLDH* lower lumbar disc herniation, *BMI* body mass index, *WSV* wedge-shaped vertebrae, *WAV* wedging angle of the vertebrae, *TL* thoracolumbar kyphotic angle, *LL* lumbar lordosis

^a^Independent *t* test

^b^Chi-square tests

^*^P < 0.05

**Table 2:** Predictive factors for ULDH: multiple logistic regression analysis

Variable	*B* value	Wald	*P* value	OR value	95% CI
WAV	− 0.244	13.341	< 0.001^*^	0.783	0.687–0.893
TL	−0.186	11.358	0.001^*^	0.831	0.746–0.925

*ULDH* upper lumbar disc herniation, *OR* odds ratio, *CI* confidence interval, *WAV* wedging angle of the vertebrae, *TL* thoracolumbar kyphotic angle

^*^P < 0.05

**Table 3:** Sensitivity, specificity, AUC, and cutoff of predictive factors for ULDH

Variable	Sensitivity	Specificity	AUC	Cutoff	*P* value
WAV	0.815	0.785	0.868	5.35	< 0.001^*^
TL	0.862	0.738	0.880	8.35	< 0.001^*^

*AUC* area under the curve, *ULDH* upper lumbar disc herniation, *WAV* wedging angle of the vertebrae, *TL* thoracolumbar kyphotic angle

^*^P < 0.05

**Table 4:** Differences in the incidence of ULDH in patient with 0, 1, or 2 predictive factors

Predictor	OR	95% CI	*P* value
0	1		
1	1.513	0.728–3.145	0.267
2	3.667	1.588–8.466	0.002^*^

*ULDH* upper lumbar disc herniation, *OR* odds ratio, *CI* confidence interval

^*^P < 0.05

## Discussion

So far, there is some confusion about the levels of ULDH. Although some literature also included the L3/4 and T12/L1 disc levels into ULDH [1, 7–10], the general consensus considers only L1/2 and L2/3, as does this current study, as ULDH. Many studies have demonstrated that the development of LDH may be influenced by several factors, including the sex, age, trauma, smoking history, chronic cough, obesity, chronic degeneration, and kyphosis [11–13]. However, because of the rarity of ULDH, its pathogenesis has not been thoroughly studied.

In clinical practice, we noted that the ULDH patients visiting our institution had one significant radiologic feature which is WSV. Moreover, some previous authors have been performed to discuss the function of the WSV contributing to ULDH [6, 7]. However, Wu et al. [8] proposed that there are no significant correlative analyses between isolated ULDH and adjacent WSV. In the present study, the incidence of WSV was detected in 44.6% (29/65) of ULDH patients treated, and the average WAV was 11.2°, which were significantly different from the LLDH group; these findings are similar to Xu et al.’s study [6]. We further found that the WSV is an independent risk factor for ULDH, and multivariate logistic regression analysis and cutoff values have shown that the existence of two factors (WAV > 5.35° and TL > 8.35°) was significantly correlated with ULDH. How does WSV affect the formation of ULDH? Firstly, we believed that the WSV can increase the shear and compressive forces of adjacent segments by changing the angle of endplates, thereby accelerating the degeneration of adjacent intervertebral discs and even leading to disc herniation [6, 14–16]. Secondly, WSV contributes greatly to the composition of thoracolumbar kyphosis, which is prone to local kyphosis. At present, the relationship between ULDH and local kyphosis remains inconclusive. But Bradford and Garica [17] and Leroux et al. [18] believed that when the kyphosis deformity occurs, the relative local weight-bearing line of the spine moves forward, the pressure on the front of the intervertebral disc increases, and the traction tension on the back increases, which makes the posterior annulus of the intervertebral disc prone to tear, leading to or accelerating the herniation of the intervertebral disc. In our current study, we found the patients with WAV > 5.35° and TL > 8.35° were more likely to suffer ULDH. Finally, previous studies have suggested that the wedge deformation of vertebral body may be related to endplate injury [6, 7, 19, 20]. And the endplate injury is also considered to be one of the main causes of disc degeneration [20–23]. In the process of injury, the integrity of the endplate was impaired, the blood supply to the intervertebral disc was affected, and its nutritional pathway was damaged, which eventually leads to the degeneration of the intervertebral disc and even the herniated disc. Consequently, from the findings of this study, it should be noted that the patients with vertebral wedge deformation combined with thoracolumbar kyphosis have a higher risk of ULDH.

However, there are some limitations to this retrospective study. The number of ULDH in this study is relatively low because of rarity of its incidence. There may be a selection bias resulting in this finding. And there is still a need for a large sample multicenter study to further confirm this result. In addition, many other factors leading to disc herniation need to be investigated in future studies for more accurate evaluation.

## Conclusion

In our study, the incidence of WSV was detected in 44.6% of ULDH patients treated, and the average WAV was 11.2°. We further found that the WSV is an independent risk factor for ULDH, and multivariate logistic regression analysis and cutoff values have shown that the existence of two factors (WAV > 5.35° and TL > 8.35°) was significantly correlated with ULDH. We should recognize that patients with vertebral wedge deformation and thoracolumbar kyphosis have a higher risk of ULDH.

## Data Availability

Data requests are available from the corresponding author.

## Abbreviations

AUC: Area under the curve
BMI: Body mass index
CI: Confidence interval
LL: Lumbar lordosis
LLDH: Lower lumbar disc herniation
OR: Odds ratio
PELD: Percutaneous endoscopic lumbar discectomy
PLIF: Posterior lumbar interbody fusion
TL: Thoracolumbar kyphotic angle
ULDH: Upper lumbar disc herniation
WAV: Wedging angle of the vertebrae
WSV: Wedge-shaped vertebrae

## References

[1] Sanderson SP, Houten J, Errico T, Forshaw D, Bauman J, Cooper PR (2004). The unique characteristics of “upper” lumbar disc herniations. Neurosurgery.

[2] Albert TJ, Balderston RA, Heller JG, Herkowitz HN, Garfin SR, Tomany K, An HS, Simeone FA (1993). Upper lumbar disc herniations. J Spinal Disord.

[3] Kortelainen P, Puranen J, Koivisto E, Lahde S (1985). Symptoms and signs of sciatica and their relation to the localization of the lumbar disc herniation. Spine J.

[4] Abdullah AF, Ditto EW, Byrd EB, Williams R (1974). Extreme lateral lumbar disc herniations: clinical syndrome and special problems of diagnosis. J Neurosurg.

[5] Ido K, Shimizu K, Tada H, Matsuda Y, Shikata J, Nakamura T (1998). Considerations for treatment of patients with upper lumbar disc herniations. J Spinal Disord.

[6] Xu JX, Yang SD, Wang BL, Yang DL, Ding WY, Shen Y (2015). Correlative analyses of isolated upper lumbar disc herniation and adjacent wedge-shaped vertebrae. Int J Clin Exp Med.

[7] Liu N, Chen ZQ, Qi Q, Shi ZF (2014). The relationship of symptomatic thoracolumbar disc herniation and Scheuermann’s disease. Eur Spine J.

[8] Wu JL, Zhang C, Zheng WJ, Hong CS, Li CQ, Zhou Y (2016). Analysis of the characteristics and clinical outcomes of percutaneous endoscopic lumbar discectomy for upper lumbar disc herniation. World Neurosurg.

[9] Son S, Lee SG, Kim WK, Ahn Y (2018). Advantages of a microsurgical translaminar approach (keyhole laminotomy) for upper lumbar disc herniation. World Neurosurg.

[10] Jha RT, Syed HR, Catalino M, Sandhu FA (2017). Contralateral approach for minimally invasive treatment of upper lumbar intervertebral disc herniation: technical note and case series. World Neurosurg.

[11] Helivaara M, Knekt P, Aromaa A (1987). Incidence and risk factors of herniated lumbar intervertebral disc or sciatica leading to hospitalization. J Clin Epidemiol.

[12] Fotakopoulos G, Makris D, Kotlia P, Tzerefos C, Fountas K (2018). Recurrence is associated with body mass index in patients undergoing a single-level lumbar disc herniation surgery. J Clin Med Res.

[13] Ohnishi K, Miyamoto K, Kanamori Y, Kodama H, Hosoe H, Shimizu K (2005). Anterior decompression and fusion for multiple thoracic disc herniation. J Bone Joint Surg (Br).

[14] Briggs AM, Wrigley TV, van Dieën JH, Phillips B, Lo SK, Greig AM, Bennell KL (2006). The effect of osteoporotic vertebral fracture on predicted spinal loads in vivo. Eur Spine J.

[15] Aquarius R, Homminga J, Verdonschot N, Tanck E (2011). The fracture risk of adjacent vertebrae is increased by the changed loading direction after a wedge fracture. Spine (Phila Pa 1976).

[16] Michalek AJ, Buckley MR, Bonassar LJ, Cohen I, Iatridis JC (2009). Measurement of local strains in intervertebral disc anulus fibrosus tissue under dynamic shear: contributions of matrix fiber orientation and elastin content. J Biomech.

[17] Bradford DS, Garica A (1969). Neurological complications in Scheuermann’s disease: a case report and review of the literature. J Bone Joint Surg Am.

[18] Leroux JL, Fuentes JM, Baixas P, Benezech J, Chertok P, Blotman F (1992). Lumbar posterior marginal node (LPMN) in adults. Report of fifteen cases. Spine J.

[19] Paajanen H, Alanen A, Erkintalo M, Salminen JJ, Katevuo K (1989). Disc degeneration in Scheuermann disease. Skelet Radiol.

[20] Dolan P, Luo J, Pollintine P, Landham PR, Stefanakis M, Adams MA (2013). Intervertebral disc decompression following endplate damage: implications for disc degeneration depend on spinal level and age. Spine (Phila Pa 1976).

[21] Kerttula LI, Serlo WS, Tervonen OA, Paakko EL, Vanharanta HV (2000). Post-traumatic findings of the spine after earlier vertebral fracture in young patients: clinical and MRI study. Spine J.

[22] Määttä JH, Kraatari M, Wolber L, Niinimäki J, Wadge S, Karppinen J, Williams FM (2014). Vertebral endplate change as a feature of intervertebral disc degeneration: a heritability study. Eur Spine J.

[23] Urban JP, Smith S, Fairbank JC (2004). Nutrition of the intervertebral disc. Spine (Phila Pa 1976).

